# Supplementary material for: Higher Resistance of Yersinia enterocolitica in Comparison to Yersinia pseudotuberculosis to Antibiotics and Cinnamon, Oregano and Thyme Essential Oils
Source: Pathogens. 2022 Dec 1;11(12):1456. doi: 10.3390/pathogens11121456 (PMC9784965; doi:10.3390/pathogens11121456)
Supplement: Supplementary file 1 [file pathogens-11-01456-s001.zip › Figure S1 EOs composition.pdf]

ANALYTICAL CERTIFICATE

NAME: ESSENTIAL OIL CINNAMON

CODE: E0074

INCI: Cinnamomum Zeylanicum Bark Oil

DENSITY (20 °C)	1.034 g/cm^3
REFRACTIVE INDEX (20 °C)	1.576
OPTICAL ROTATION (20 °C)	−2°

**COMPONENT**	**%**
1,8-Cineole (Eucalyptol)	0.411
Beta-caryophyllene	4.853
Cinnamyl alcohol	0.157
Eugenol	18.205
Limonene	0.175
Linalool	2.073
Para-Cymen	0.395
Trans-cinnamaldehyde	65.043

Nobilis Tilia, s.r.o.
Vlčí Hora 147, 407 46 Krásná Lípa
Tel.: +420 412 383 421
Fax: +420 412 383 899
E-mail: nobilis@nobilis.cz
Web: www.nobilis.cz

ANALYTICAL CERTIFICATE

NAME: ESSENTIAL OIL OREGANO

CODE: E0110

INCI: Origanum Vulgaris Oil

DENSITY (20 °C)	0.948 g/cm^3
REFRACTIVE INDEX (20 °C)	1.508
OPTICAL ROTATION (20 °C)	0°

**COMPONENT**	**%**
Alpha-Pinene	0.776
Alpha-Terpinene	1.186
Alpha-Thujene	1.516
Beta-caryophyllene	2.141
Beta-Pinene	0.200
Borneol	0.217
Gamma-Terpinene	6.026
Camphen	0.237
Carvacrol	73.561
Limonene	0.331
Linalool	0.100
Myrcen	2.161
Para-Cymen	6.972
Terpin-1-en-4-ol	0.788

ANALYTICAL CERTIFICATE

NAME:	ESSENTIAL OIL THYME
CODE:	E0069
INCI:	Thymus Vulgaris Oil

DENSITY (20 °C)	0.922 g/cm^3
REFRACTIVE INDEX (20 °C)	1.495
OPTICAL ROTATION (20 °C)	-1°

**COMPONENT**	**%**
1,8-Cineole	0.243
Alpha-Pinene	1.198
Alpha-Terpinene	1.824
Alpha-Terpineneol	0.224
Alpha-Thujene	0.888
Beta-caryophyllene	3.587
Beta-Pinene	0.200
Borneol	1.083
Gamma-Terpinene	12.216
Camphen	0.786
Camphor	0.649
Carvacrol	2.413
Carvone	0.256
Limonene	0.428
Linalool	3.047
Myrcen	2.082
Para-Cymen	17.668
Terpin-1-en-4-ol	1.399
Thymol	45.847

Nobilis Tilia, s.r.o.
Vlčí Hora 147, 407 46 Krásná Lípa
Tel.: +420 412 383 421
Fax: +420 412 383 899
E-mail: nobilis@nobilis.cz
Web: www.nobilis.cz
